# Hospital Construction

**Published:** 1896-08-29

**Authors:** 


### HOSPITAL CONSTRUCTION.

### HOMŒOPATHIC HOSPITAL, MELBOURNE.

The Homœopathic Hospital at Melbourne, of which the first wing was opened some ten years since and the last addition completed in 1890, is a fine building, with accommodation for 80 beds. A special architectural feature consists in the large balconies running all round the building, made pleasant convalescent resorts with lounges, plants, &c., and reached from the wards by French windows, so as to allow of the easy transport of patients, bed and all, if so desired, into the fresh air.

Entering the hospital the homœopathic motto, "Similia similibus curantur" (like things are cured by like), is seen writ large on an arch within the door, and a stained-glass window, given by one of the medical staff, Dr. Ray, in memory of his father (himself one of the first members of the staff), beautifies the staircase, the subject figure being Æsculapius. The wards are bright and cheerful enough with their well-polished floors and abundance of flowers—special wardsmen, by the bye, are detailed for the care of the floors—and a pleasant outlook there is over the small, but carefully laid out and kept garden. Pictures grace the walls, and a member of the nursing staff with artistic tastes has contributed her share of ornamentation by painting dainty pictures on the ventilators. Both medical and surgical cases, male and female, are taken in at the Homœopathic Hospital, and the admission for poor patients is free, while there are also private wards for the benefit of those better off people who may like to put themselves under treatment there at the rate of two to three guineas a week.

In one corner of the general surgical ward is an endowed cot, which costs £35 a year to maintain. It is under the fostering care of the Ladies' Aid Association, members of which make themselves responsible for its support, and also visit the hospital regularly, giving much valuable help. Each ward is provided with a small kitchen or pantry, fitted with gas-stove, &c., and also a room for the nurses.

The nursing staff are well looked after. A training school is attached to the hospital, and there is also a private nursing institution. The nurses' quarters are on the top floor of the building, and are very comfortable, including a dining-hall and sitting-room, with a good library, for which latter contributions are received gladly. The period of training is for three years, during which very complete courses of lectures are given by the various members of the medical staff. These lectures are popular with outsiders, who are permitted to attend them by ticket, and are always ready to avail themselves of the privilege. A printed list of "Don'ts, for Hospital Nurses," by Dr. Bouton, distributed amongst the nurses is a useful little publication, giving much practical information in small compass. Miss Campbell, the present matron, has held that post for ten years past, and was the first nurse trained at the hospital. She now has under her a staff of thirty-one nurses, including probationers.

Many people have an idea that a homœopathic hospital must contain only those undergoing homœopathic treatment by means of the little globules most generally associated with the word by the lay public, and that surgery plays but a small part in the work of such an institution. But this hospital contains surgical wards in which are many serious cases, and during the year 1894 there were some 300 major operations, a large proportion being successful. The dressings used are the same as those in vogue in other hospitals, but the drugs for internal medication are of course given after the homœopathic method.

There is an out-patient department and dispensary (the latter in the basement of the building), quarters for the servants, and a laundry. The secretary and superintendent, Mr. Bennett, has held that position since the starting of the hospital, and is universally popular. There is an air of general wellbeing and comfort about the whole institution which testifies to its internal good management. Much interest is taken in its affairs by the president, Sir W. J. Clarke, and practical proof of the measure of support which it obtains may be observed by a glance at the list of subscribers and donors of considerable sums inscribed on a marble slab in the entrance hall.

